# (3-Chloro­phen­yl)[(*E*)-2-(1,3-di­thio­lan-2-yl­idene)hydrazinyl­idene]methyl 3-chloro­benzoate

**DOI:** 10.1107/S1600536813009239

**Published:** 2013-04-13

**Authors:** Ling Yin

**Affiliations:** a Department of Chemistry and Chemical Engineering, Jining University, Qufu 273155, People’s Republic of China

## Abstract

In the title compound, C_17_H_12_Cl_2_N_2_O_2_S_2_, the di­thia­cyclo­pentane ring has an envelope conformation with one of the methyl­ene C atoms as the flap. The chloro­phenyl rings make a dihedral angle of 82.63 (7)°. In the crystal, π–π inter­actions between the benzene rings of neighbouring mol­ecules [centroid–centroid distance = 3.547 (2) Å] link the mol­ecules into inversion dimers. Weak non-classical C—H⋯*X* (*X* = O, N, Cl) inter­actions further consolidate the packing, forming a layer structure parallel to (110).

## Related literature

For applications of heterocyclic di­thiol­ane compounds, see: Tanaka *et al.* (1976 ▶); Wang *et al.* (1994 ▶). For the crystal structure of (*E*)-[2-(1,3-di­thio­lan-2-yl­idene)hydrazinyl­idene](3-fluoro­phen­yl)methyl 3-fluoro­benzoate, see: Yin (2013 ▶).
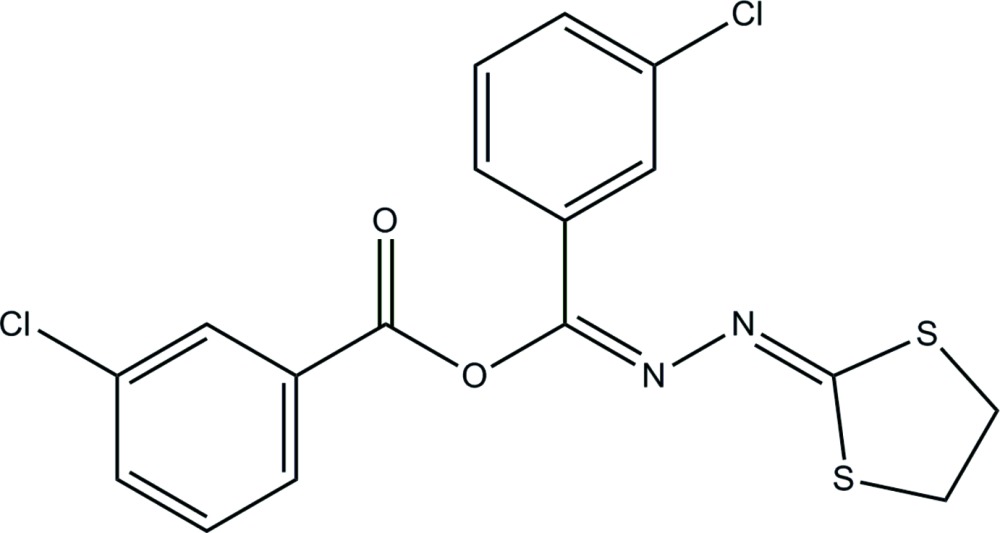


## Experimental

### 

#### Crystal data

C_17_H_12_Cl_2_N_2_O_2_S_2_
*M* *_r_* = 411.31
Triclinic, 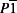

*a* = 8.960 (5) Å
*b* = 9.944 (6) Å
*c* = 11.128 (6) Å
α = 104.174 (8)°
β = 111.041 (7)°
γ = 99.410 (2)°
*V* = 861.9 (8) Å^3^
*Z* = 2
Mo *K*α radiation
μ = 0.63 mm^−1^
*T* = 113 K
0.34 × 0.25 × 0.20 mm

#### Data collection

Rigaku Saturn CCD area-detector diffractometer
Absorption correction: multi-scan (*CrystalClear-SM Expert*; Rigaku/MSC, 2009 ▶) *T*_min_ = 0.884, *T*_max_ = 0.884
11088 measured reflections
4080 independent reflections
3115 reflections with *I* > 2σ(*I*)
*R*_int_ = 0.034

#### Refinement

*R*[*F* ^2^ > 2σ(*F* ^2^)] = 0.030
*wR*(*F* ^2^) = 0.074
*S* = 0.95
4080 reflections
226 parameters
H-atom parameters constrained
Δρ_max_ = 0.46 e Å^−3^
Δρ_min_ = −0.22 e Å^−3^

### 

Data collection: *CrystalClear-SM Expert* (Rigaku/MSC, 2009 ▶); cell refinement: *CrystalClear-SM Expert*; data reduction: *CrystalClear-SM Expert*; program(s) used to solve structure: *SHELXS97* (Sheldrick, 2008 ▶); program(s) used to refine structure: *SHELXL97* (Sheldrick, 2008 ▶); molecular graphics: *SHELXTL* (Sheldrick, 2008 ▶); software used to prepare material for publication: *SHELXTL*.

## Supplementary Material

Crystal structure: contains datablock(s) I, global. DOI: 10.1107/S1600536813009239/cv5400sup1.cif

Structure factors: contains datablock(s) I. DOI: 10.1107/S1600536813009239/cv5400Isup2.hkl

Supplementary material file. DOI: 10.1107/S1600536813009239/cv5400Isup3.cml

Additional supplementary materials:  crystallographic information; 3D view; checkCIF report

## Figures and Tables

**Table 1:** Hydrogen-bond geometry (Å, °)

*D*—H⋯*A*	*D*—H	H⋯*A*	*D*⋯*A*	*D*—H⋯*A*
C10—H10*A*⋯N1^i^	0.95	2.59	3.534 (2)	173
C14—H14*A*⋯Cl1^ii^	0.95	2.80	3.727 (2)	165
C16—H16*B*⋯O2^iii^	0.99	2.48	3.274 (3)	137

Symmetry codes: (i) 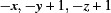; (ii) 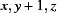; (iii) 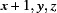.

**Fig. 1.**
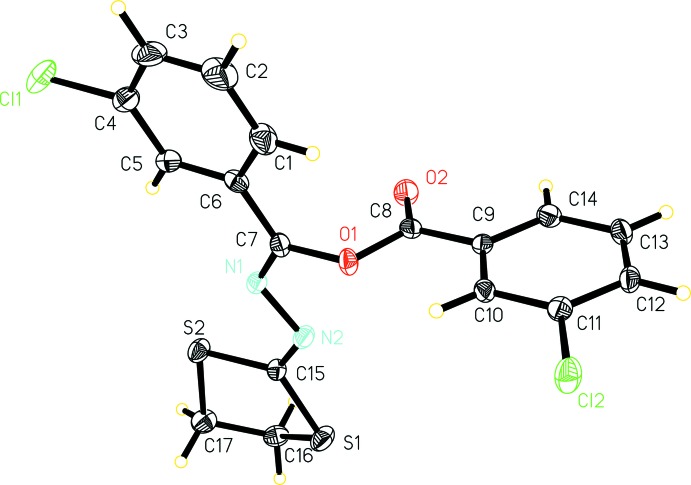
View of the title compound showing the atomic numbering and 50% probability displacement ellipsoids.

## supplementary crystallographic information

### Comment

Many dithiolan heterocyclic compounds have been widely used as potent and broad-spectrum fungicides (Tanaka *et al.*, 1976; Wang *et al.*, 1994). In order to search for new heterocylic compounds with higher biological activities, we synthesized the (*E*)-((1,3-dithiolan-2-yl)diazenyl)(3-chlorophenyl)methyl 3-chlorobenzoate (I) and described its structure here.

In (I) (Fig. 1), the dithiacyclopentane ring has an envelope conformation with C16 atom as a flap. Two chlorophenyl rings (C1—C6 and C9—C14) in the molecule form a dihedral angle of 82.63 (7)°. All bond lengths and angles are normal and in a good agreement with those reported previously for related compounds (Yin, 2013)

In the crystal, π-π interactions between the benzene rings from two neighbouring molecules [centroid-centroid distance of 3.547 (2) Å] link the latters into centrosymmetric dimer, and weak non-classical C—H···*X* (*X*=O, N, Cl) interactions (Table 1) consolidate further the packing.

### Experimental

1.34 g (10 mmol) of (1,3-dithiolan-2-ylidene)hydrazine and 20 mmol triethylamine was dissolved in 15 ml of dichloromethane and stirred at room temperature, 3.50 g (20 mmol) 3-chlorobenzoyl chloride was added dropwise to the mixture. The reaction mixture was stirred vigorously at 0 centigrade for 4 h. The reaction mixture was poured into 200 ml of water and extracted with three 50-ml portions of dichloromethane. The combined extracts were washed with saturated brine, dried over anhydrous sodium sulfate and evaporated on a rotary evaporator to afford the crude product, which was purified by column chromatography to yield the pure product as colorless crystals. Single crystals suitable for X-ray diffraction were obtained through slow evaporation of a solution of the pure title compound in ethanol.

### Refinement

All H atoms bonded on carbon were found on difference maps, with C–H = 0.93 or 0.97 Å, and included in the final cycles of refinement using a riding model, with *U*_iso_(H) = 1.2*U*_eq_(C).

### Crystal data

**Table:**

C_17_H_12_Cl_2_N_2_O_2_S_2_	*Z* = 2
*M**_r_* = 411.31	*F*(000) = 420
Triclinic, *P*1	*D*_x_ = 1.585 Mg m^−^^3^
Hall symbol: -P 1	Mo *K*α radiation, λ = 0.71073 Å
*a* = 8.960 (5) Å	Cell parameters from 3010 reflections
*b* = 9.944 (6) Å	θ = 2.1–27.9°
*c* = 11.128 (6) Å	µ = 0.63 mm^−^^1^
α = 104.174 (8)°	*T* = 113 K
β = 111.041 (7)°	Block, colourless
γ = 99.410 (2)°	0.34 × 0.25 × 0.20 mm
*V* = 861.9 (8) Å^3^	

### Data collection

**Table:**

Rigaku Saturn CCD area-detector diffractometer	4080 independent reflections
Radiation source: rotating anode	3115 reflections with *I* > 2σ(*I*)
Multilayer monochromator	*R*_int_ = 0.034
Detector resolution: 14.63 pixels mm^-1^	θ_max_ = 27.9°, θ_min_ = 2.1°
ω and φ scans	*h* = −11→11
Absorption correction: multi-scan (*CrystalClear-SM Expert*; Rigaku/MSC, 2009)	*k* = −13→13
*T*_min_ = 0.884, *T*_max_ = 0.884	*l* = −14→14
11088 measured reflections	

### Refinement

**Table:**

Refinement on *F*^2^	Primary atom site location: structure-invariant direct methods
Least-squares matrix: full	Secondary atom site location: difference Fourier map
*R*[*F*^2^ > 2σ(*F*^2^)] = 0.030	Hydrogen site location: inferred from neighbouring sites
*wR*(*F*^2^) = 0.074	H-atom parameters constrained
*S* = 0.95	*w* = 1/[σ^2^(*F*_o_^2^) + (0.0343*P*)^2^] where *P* = (*F*_o_^2^ + 2*F*_c_^2^)/3
4080 reflections	(Δ/σ)_max_ = 0.001
226 parameters	Δρ_max_ = 0.46 e Å^−^^3^
0 restraints	Δρ_min_ = −0.22 e Å^−^^3^

### Special details

**Table:**

Geometry. All e.s.d.'s (except the e.s.d. in the dihedral angle between two l.s. planes) are estimated using the full covariance matrix. The cell e.s.d.'s are taken into account individually in the estimation of e.s.d.'s in distances, angles and torsion angles; correlations between e.s.d.'s in cell parameters are only used when they are defined by crystal symmetry. An approximate (isotropic) treatment of cell e.s.d.'s is used for estimating e.s.d.'s involving l.s. planes.
Refinement. Refinement of *F*^2^ against ALL reflections. The weighted *R*-factor *wR* and goodness of fit *S* are based on *F*^2^, conventional *R*-factors *R* are based on *F*, with *F* set to zero for negative *F*^2^. The threshold expression of *F*^2^ > σ(*F*^2^) is used only for calculating *R*-factors(gt) *etc*. and is not relevant to the choice of reflections for refinement. *R*-factors based on *F*^2^ are statistically about twice as large as those based on *F*, and *R*- factors based on ALL data will be even larger.

### Fractional atomic coordinates and isotropic or equivalent isotropic displacement parameters (Å^2^)

**Table:**

	*x*	*y*	*z*	*U*_iso_*/*U*_eq_	
S1	0.30877 (5)	0.64714 (4)	0.23284 (4)	0.02513 (11)	
S2	0.09584 (5)	0.34521 (4)	0.09623 (4)	0.02211 (11)	
Cl1	−0.52910 (5)	−0.06840 (4)	0.12078 (4)	0.03188 (12)	
Cl2	0.10357 (5)	1.00841 (4)	0.89309 (4)	0.02569 (11)	
O1	−0.11886 (13)	0.59701 (10)	0.42806 (10)	0.0180 (2)	
O2	−0.27715 (13)	0.66856 (11)	0.26079 (10)	0.0234 (3)	
N1	−0.07149 (15)	0.43373 (13)	0.26298 (12)	0.0168 (3)	
N2	0.05526 (15)	0.55241 (13)	0.28369 (13)	0.0203 (3)	
C1	−0.3769 (2)	0.38581 (18)	0.40729 (17)	0.0269 (4)	
H1B	−0.3438	0.4807	0.4699	0.032*	
C2	−0.5067 (2)	0.2817 (2)	0.39886 (19)	0.0356 (4)	
H2B	−0.5629	0.3060	0.4555	0.043*	
C3	−0.5555 (2)	0.1434 (2)	0.30938 (18)	0.0320 (4)	
H3A	−0.6454	0.0725	0.3034	0.038*	
C4	−0.47179 (19)	0.10906 (17)	0.22822 (15)	0.0222 (4)	
C5	−0.34232 (18)	0.21123 (16)	0.23301 (14)	0.0180 (3)	
H5A	−0.2872	0.1863	0.1756	0.022*	
C6	−0.29455 (18)	0.35118 (16)	0.32354 (15)	0.0173 (3)	
C7	−0.15643 (18)	0.46156 (15)	0.33172 (14)	0.0163 (3)	
C8	−0.19326 (17)	0.69437 (16)	0.37966 (15)	0.0167 (3)	
C9	−0.15680 (17)	0.82978 (15)	0.49075 (14)	0.0156 (3)	
C10	−0.05798 (17)	0.84909 (15)	0.62690 (15)	0.0162 (3)	
H10A	−0.0168	0.7734	0.6523	0.019*	
C11	−0.02184 (18)	0.98142 (16)	0.72356 (14)	0.0172 (3)	
C12	−0.08166 (18)	1.09324 (16)	0.68971 (15)	0.0193 (3)	
H12A	−0.0540	1.1835	0.7580	0.023*	
C13	−0.18269 (19)	1.07119 (16)	0.55451 (16)	0.0213 (3)	
H13A	−0.2265	1.1462	0.5300	0.026*	
C14	−0.21989 (18)	0.94018 (16)	0.45513 (16)	0.0195 (3)	
H14A	−0.2885	0.9257	0.3626	0.023*	
C15	0.13750 (18)	0.51590 (16)	0.21249 (15)	0.0172 (3)	
C16	0.31996 (19)	0.54981 (17)	0.07793 (15)	0.0225 (4)	
H16A	0.2393	0.5664	−0.0013	0.027*	
H16B	0.4331	0.5832	0.0830	0.027*	
C17	0.27909 (19)	0.39074 (17)	0.06257 (16)	0.0246 (4)	
H17A	0.3744	0.3703	0.1278	0.030*	
H17B	0.2568	0.3319	−0.0310	0.030*	

### Atomic displacement parameters (Å^2^)

**Table:**

	*U*^11^	*U*^22^	*U*^33^	*U*^12^	*U*^13^	*U*^23^
S1	0.0235 (2)	0.0184 (2)	0.0313 (2)	−0.00078 (17)	0.01585 (19)	0.00201 (18)
S2	0.0228 (2)	0.0166 (2)	0.0255 (2)	0.00214 (17)	0.01373 (18)	0.00125 (17)
Cl1	0.0338 (2)	0.0226 (2)	0.0255 (2)	−0.00746 (19)	0.00331 (19)	0.00860 (18)
Cl2	0.0353 (2)	0.0202 (2)	0.0180 (2)	0.00879 (18)	0.00931 (17)	0.00234 (16)
O1	0.0231 (6)	0.0137 (5)	0.0157 (5)	0.0080 (5)	0.0067 (4)	0.0024 (4)
O2	0.0237 (6)	0.0210 (6)	0.0187 (6)	0.0067 (5)	0.0025 (5)	0.0042 (5)
N1	0.0171 (6)	0.0139 (6)	0.0194 (6)	0.0033 (5)	0.0083 (5)	0.0053 (5)
N2	0.0192 (7)	0.0143 (7)	0.0258 (7)	0.0013 (5)	0.0111 (6)	0.0037 (6)
C1	0.0310 (10)	0.0262 (9)	0.0335 (9)	0.0128 (8)	0.0210 (8)	0.0116 (8)
C2	0.0345 (10)	0.0436 (11)	0.0494 (12)	0.0182 (9)	0.0324 (10)	0.0225 (10)
C3	0.0201 (9)	0.0381 (11)	0.0453 (11)	0.0056 (8)	0.0169 (8)	0.0231 (9)
C4	0.0191 (8)	0.0230 (9)	0.0210 (8)	0.0025 (7)	0.0033 (7)	0.0111 (7)
C5	0.0162 (8)	0.0217 (8)	0.0170 (7)	0.0044 (7)	0.0066 (6)	0.0087 (7)
C6	0.0166 (7)	0.0220 (8)	0.0177 (8)	0.0089 (7)	0.0080 (6)	0.0100 (6)
C7	0.0192 (8)	0.0147 (8)	0.0138 (7)	0.0068 (6)	0.0050 (6)	0.0042 (6)
C8	0.0135 (7)	0.0167 (8)	0.0221 (8)	0.0047 (6)	0.0093 (6)	0.0071 (6)
C9	0.0130 (7)	0.0135 (7)	0.0205 (8)	0.0028 (6)	0.0086 (6)	0.0039 (6)
C10	0.0160 (7)	0.0138 (7)	0.0218 (8)	0.0050 (6)	0.0106 (6)	0.0061 (6)
C11	0.0170 (8)	0.0174 (8)	0.0190 (8)	0.0036 (6)	0.0105 (6)	0.0051 (6)
C12	0.0208 (8)	0.0132 (8)	0.0260 (8)	0.0044 (6)	0.0143 (7)	0.0030 (6)
C13	0.0214 (8)	0.0162 (8)	0.0305 (9)	0.0086 (7)	0.0126 (7)	0.0098 (7)
C14	0.0172 (8)	0.0205 (8)	0.0208 (8)	0.0060 (7)	0.0072 (6)	0.0074 (7)
C15	0.0177 (8)	0.0140 (7)	0.0192 (8)	0.0045 (6)	0.0067 (6)	0.0060 (6)
C16	0.0186 (8)	0.0263 (9)	0.0236 (8)	0.0035 (7)	0.0109 (7)	0.0084 (7)
C17	0.0228 (9)	0.0259 (9)	0.0257 (9)	0.0050 (7)	0.0139 (7)	0.0047 (7)

### Geometric parameters (Å, º)

**Table:**

S1—C15	1.7493 (17)	C5—C6	1.394 (2)
S1—C16	1.8048 (18)	C5—H5A	0.9500
S2—C15	1.7507 (17)	C6—C7	1.473 (2)
S2—C17	1.8228 (18)	C8—C9	1.483 (2)
Cl1—C4	1.7427 (18)	C9—C14	1.392 (2)
Cl2—C11	1.7393 (17)	C9—C10	1.398 (2)
O1—C8	1.3713 (17)	C10—C11	1.382 (2)
O1—C7	1.4008 (17)	C10—H10A	0.9500
O2—C8	1.1989 (18)	C11—C12	1.386 (2)
N1—C7	1.2744 (19)	C12—C13	1.388 (2)
N1—N2	1.4058 (18)	C12—H12A	0.9500
N2—C15	1.2917 (19)	C13—C14	1.385 (2)
C1—C2	1.383 (2)	C13—H13A	0.9500
C1—C6	1.397 (2)	C14—H14A	0.9500
C1—H1B	0.9500	C16—C17	1.516 (2)
C2—C3	1.376 (3)	C16—H16A	0.9900
C2—H2B	0.9500	C16—H16B	0.9900
C3—C4	1.384 (2)	C17—H17A	0.9900
C3—H3A	0.9500	C17—H17B	0.9900
C4—C5	1.387 (2)		
			
C15—S1—C16	94.86 (8)	C10—C9—C8	121.62 (13)
C15—S2—C17	95.15 (7)	C11—C10—C9	118.14 (13)
C8—O1—C7	115.97 (11)	C11—C10—H10A	120.9
C7—N1—N2	114.80 (13)	C9—C10—H10A	120.9
C15—N2—N1	111.40 (13)	C10—C11—C12	122.18 (14)
C2—C1—C6	119.91 (16)	C10—C11—Cl2	118.80 (11)
C2—C1—H1B	120.0	C12—C11—Cl2	119.01 (12)
C6—C1—H1B	120.0	C11—C12—C13	118.94 (14)
C3—C2—C1	120.83 (16)	C11—C12—H12A	120.5
C3—C2—H2B	119.6	C13—C12—H12A	120.5
C1—C2—H2B	119.6	C14—C13—C12	120.22 (14)
C2—C3—C4	119.02 (16)	C14—C13—H13A	119.9
C2—C3—H3A	120.5	C12—C13—H13A	119.9
C4—C3—H3A	120.5	C13—C14—C9	120.04 (14)
C3—C4—C5	121.63 (16)	C13—C14—H14A	120.0
C3—C4—Cl1	118.48 (13)	C9—C14—H14A	120.0
C5—C4—Cl1	119.87 (13)	N2—C15—S1	118.01 (12)
C4—C5—C6	118.83 (15)	N2—C15—S2	126.35 (12)
C4—C5—H5A	120.6	S1—C15—S2	115.64 (9)
C6—C5—H5A	120.6	C17—C16—S1	107.62 (11)
C5—C6—C1	119.77 (14)	C17—C16—H16A	110.2
C5—C6—C7	120.01 (13)	S1—C16—H16A	110.2
C1—C6—C7	120.21 (14)	C17—C16—H16B	110.2
N1—C7—O1	122.71 (13)	S1—C16—H16B	110.2
N1—C7—C6	122.70 (14)	H16A—C16—H16B	108.5
O1—C7—C6	114.45 (13)	C16—C17—S2	108.49 (11)
O2—C8—O1	122.10 (14)	C16—C17—H17A	110.0
O2—C8—C9	125.99 (14)	S2—C17—H17A	110.0
O1—C8—C9	111.91 (12)	C16—C17—H17B	110.0
C14—C9—C10	120.46 (13)	S2—C17—H17B	110.0
C14—C9—C8	117.88 (13)	H17A—C17—H17B	108.4
			
C7—N1—N2—C15	179.07 (13)	O1—C8—C9—C14	177.72 (12)
C6—C1—C2—C3	0.5 (3)	O2—C8—C9—C10	179.57 (14)
C1—C2—C3—C4	0.5 (3)	O1—C8—C9—C10	−0.29 (19)
C2—C3—C4—C5	−1.3 (2)	C14—C9—C10—C11	−1.6 (2)
C2—C3—C4—Cl1	177.27 (12)	C8—C9—C10—C11	176.34 (13)
C3—C4—C5—C6	0.9 (2)	C9—C10—C11—C12	0.7 (2)
Cl1—C4—C5—C6	−177.58 (10)	C9—C10—C11—Cl2	−179.01 (11)
C4—C5—C6—C1	0.1 (2)	C10—C11—C12—C13	0.7 (2)
C4—C5—C6—C7	179.44 (13)	Cl2—C11—C12—C13	−179.55 (11)
C2—C1—C6—C5	−0.8 (2)	C11—C12—C13—C14	−1.3 (2)
C2—C1—C6—C7	179.86 (14)	C12—C13—C14—C9	0.4 (2)
N2—N1—C7—O1	−4.57 (19)	C10—C9—C14—C13	1.1 (2)
N2—N1—C7—C6	179.94 (12)	C8—C9—C14—C13	−176.96 (13)
C8—O1—C7—N1	89.63 (17)	N1—N2—C15—S1	−176.17 (9)
C8—O1—C7—C6	−94.53 (15)	N1—N2—C15—S2	3.35 (19)
C5—C6—C7—N1	−3.2 (2)	C16—S1—C15—N2	−163.32 (12)
C1—C6—C7—N1	176.16 (14)	C16—S1—C15—S2	17.11 (9)
C5—C6—C7—O1	−179.00 (11)	C17—S2—C15—N2	−174.68 (14)
C1—C6—C7—O1	0.33 (19)	C17—S2—C15—S1	4.84 (9)
C7—O1—C8—O2	−4.5 (2)	C15—S1—C16—C17	−37.74 (12)
C7—O1—C8—C9	175.41 (12)	S1—C16—C17—S2	45.83 (13)
O2—C8—C9—C14	−2.4 (2)	C15—S2—C17—C16	−30.73 (12)

### Hydrogen-bond geometry (Å, º)

**Table:**

*D*—H···*A*	*D*—H	H···*A*	*D*···*A*	*D*—H···*A*
C10—H10*A*···N1^i^	0.95	2.59	3.534 (2)	173
C14—H14*A*···Cl1^ii^	0.95	2.80	3.727 (2)	165
C16—H16*B*···O2^iii^	0.99	2.48	3.274 (3)	137

Symmetry codes: (i) −*x*, −*y*+1, −*z*+1; (ii) *x*, *y*+1, *z*; (iii) *x*+1, *y*, *z*.
